# Data-driven and machine learning framework for Alfalfa yield response to long-term climate variability in the Kansas High Plains (USA)

**DOI:** 10.1038/s41598-026-55545-2

**Published:** 2026-06-04

**Authors:** Farshina Nazrul, Jiyung Kim, Sourajit Dey, Bradley Whitaker, Debjani Sihi, Doohong Min, Gaurav Jha

**Affiliations:** 1 Natural Resource Ecology Laboratory (NREL), Colorado State University, Fort Collins, Colorado, United States; 2 Department of Agronomy, Kansas State University, Manhattan, KS 66506 USA; 3 Forages Production Systems Division, National Institute of Animal Science, Rural Development Administration, Cheonan, Korea; 4 Department of Electrical & Computer Engineering, Montana State University, Bozeman, MT 59717 USA; 5 Department of Plant and Microbial Biology, Department of Crop and Soil Sciences, Plant Sciences Initiative, North Carolina State University, Raleigh, NC 27695 USA

**Keywords:** Alfalfa, Climate variables, Feature selection, Minimum Redundancy Maximum Relevance (mRMR) algorithm, Machine learning framework, Kansas high plains, Climate sciences, Ecology, Ecology, Environmental sciences, Hydrology

## Abstract

Climate extremes and declining water availability in the U.S. High Plains threaten the long-term sustainability of alfalfa production. This study presents a machine learning (ML) based modeling framework to identify and evaluate key agro-climatic predictors of alfalfa (*Medicago sativa* L.) yield across nine agricultural districts in Kansas from 1981 to 2018. A novel contribution of this study is the integration of a high-dimensional climate predictor space, long-term historical records, and district-level stratification of irrigated and rainfed systems to systematically assess spatially varying climate-yield relationships. Using a high-dimensional feature space of 117 climate-derived variables from PRISM and yield data from USDA-NASS, a Minimum Redundancy Maximum Relevance (mRMR) algorithm was applied to rank predictors, followed by forward feature selection with three non-parametric regression models. Four spatial configurations were evaluated—statewide, district-specific, irrigated, and rainfed datasets. The Kansas High Plains region, with its strong west-to-east precipitation gradient, groundwater-dependent irrigation systems, and recurrent drought exposure, offers a globally relevant testbed for understanding crop-climate interactions in semi-arid environments. Model evaluation RMSE during feature selection ranged from 0.52 to 1.14 tons/acre. Dew point temperature in August and seasonal VPD metrics emerged as dominant predictors, indicating these variables are strongly associated with late-summer yield variability. Rainfed districts prioritized drought indicators (e.g., no precipitation days, VPD), while irrigated districts showed stronger associations with thermal and humidity metrics. By explicitly accounting for climate sensitivities across management regimes and spatial scales, this framework provides a transferable approach for identifying robust, region-specific climate drivers in high-dimensional agricultural datasets.

**Supplementary Information:**

The online version contains supplementary material available at 10.1038/s41598-026-55545-2.

## Introduction

Alfalfa (*Medicago sativa* L.) is a perennial forage legume with high forage yield^1,2^, high nutritive value^1,2^, and biological nitrogen fixation^2,3^ of more than 80% of the amount required^4^. Kansas ranks as the ninth largest alfalfa-producing state in the USA in 2023, with annual alfalfa hay production exceeding 2.2 million tons from about 1.73 million acres^5^.

The sustainability of alfalfa production in the Kansas High Plains is under increasing pressure due to intensified climate extremes (severe to exceptional droughts) and long-term declines in groundwater levels from the Ogallala Aquifer^6^. The water levels have declined by 25 to 103 feet across five groundwater management districts, leading to almost a 60% reduction of the original water level in some areas since predevelopment in the 1950s^7^. The critical role of groundwater, particularly from the Ogallala Aquifer, was emphasized in sustaining crop production across the Great Plains^8^. Alfalfa yields can drop over 70% during drought^9,10^. As a result, the anticipated decline in the level of the Ogallala aquifer poses a future challenge for alfalfa cultivation in Kansas^11^. The amount and timing of precipitation during the growing season are critical for alfalfa growth, regrowth, and total biomass accumulation.

Alfalfa is typically established in late summer or early fall to reduce weed competition and favorable soil moisture in Kansas^12^. Once established, alfalfa can be harvested multiple times per growing season, often beginning in May and extending through the last week of September or the first fortnight of October, depending on precipitation and irrigation availability^13^. In Kansas, the growing season window for alfalfa differs regionally. In western Kansas, warmer spring temperatures allow green-up to begin as early as early March, while in eastern Kansas, this typically occurs later in the month. Similarly, the final harvest can be extended until the last week of October in western Kansas, whereas in eastern Kansas, the last cutting is recommended no later than the first week of October to reduce the risk of winter injury^13,14^. First cutting is usually done at 10% flowering, followed by subsequent cuttings with a 28–32 days interval, balancing forage yield with nutritive value, particularly crude protein and digestibility. Alfalfa grows well in well-drained soil but requires a lot of water, with a water demand from 26 to 40 inches. In Kansas, where water availability varies substantially across regions, alfalfa serves as a critical forage crop for both dairy and beef production, and its productivity is highly sensitive to climatic variability^11^.

To better understand and manage alfalfa growth, several key climatic variables are often considered. Precipitation directly influences soil moisture availability and irrigation demand, while temperature regulates growth rate, dormancy, and seasonal development^15^. Vapor pressure deficit (VPD) serves as a critical indicator of atmospheric water demand, influencing evapotranspiration and plant water stress, whereas dew point temperature reflects ambient moisture conditions that affect transpiration efficiency^16–18^. A high dew point temperature causes the winterkill and summer slump in winter and summer, respectively. The VPD at a given temperature can affect plant growth due to its impact on stomatal aperture, carbon dioxide (CO_2_) uptake, transpiration, or leaf temperature^19,20^. It is a useful parameter to evaluate the drought condition^21^ and water productivity^22,23^. The low VPD negatively affects plant growth and productivity^24^. Alfalfa, being a perennial crop with distinct growth cycles influenced by varying weather conditions, particularly VPD, experiences fluctuations in water productivity^25^. The optimal temperature range for alfalfa growth typically falls between 65 and 85 ℉^1^, with alfalfa capable of tolerating the maximum daily growth temperature of up to 100 ℉^26^. Together, these variables provide an integrated view of water supply and atmospheric demand, which are central to alfalfa production in irrigated and rainfed environments of Kansas.

Given the impacts of climate variability, there is growing interest in using machine learning (ML) techniques^27,28^. Recent studies have applied ML approaches to evaluate climate-driven agricultural responses and water-use dynamics, demonstrating the value of data-driven models for capturing nonlinear relationships and future climate impacts^29–32^. In parallel, several studies have also explored spatial variability in water productivity and drought resilience in alfalfa and other cropping systems using field-based and high-throughput approaches, highlighting the complexity of climate-yield interactions across management conditions^33,34^.

ML approaches such as feature selection offer tools for handling high-dimensional climate data and uncovering complex interactions that traditional statistical models may overlook in crop model simulation and crop yield predictions^29,30^. Among various feature selection techniques, the Minimum Redundancy Maximum Relevance (mRMR) algorithm has shown potential in analyzing the complex, interrelated climate variables that show high correlation with crop yield. The mRMR selects features that are both highly relevant to the target outcome (i.e., yield) and minimally redundant with one another, making it well-suited for high-dimensional environmental data^31,32^. Its ability to balance relevance and redundancy enhances the accuracy and interpretability of the associated ML models in both classification and regression tasks^33–35^. Alfalfa is an important forage crop in the Kansas High Plains, where its productivity plays an important role in both agricultural systems and regional water use. However, knowledge of how long-term climate variability has shaped alfalfa yield trends in this region remains limited. Previous studies have largely focused on short-term field experiments, region-wide modeling using a small set of climate variables, or coarse-scale assessments that do not consider individual impact on the nine agricultural districts^4,8,13,36–39^. These approaches often overlook the high dimensionality and interdependence of variables in climate datasets that are critical to understanding crop-climate interactions over time^27,28^. Moreover, only a few studies have applied statistical and data-driven feature selection techniques, such as mRMR, to identify the most informative predictors of alfalfa yield across diverse agro-climatic settings.

To address these limitations, this study presents a high-dimensional analysis of long-term climate impacts on alfalfa yield in the Kansas High Plains. In this regard, a high-dimensional feature selection strategy was integrated with an ML-based regression framework to uncover the most relevant climate predictors of alfalfa yield across both irrigated and rainfed conditions in Kansas. By integrating 117 climate-derived predictors over a 38-year period (1981–2018) and differentiating irrigated vs. rainfed systems through the individual analysis of nine agricultural districts^22,37–39^ and grouped subclasses according to the irrigation water management practices, this study addresses this key methodological and contextual gap using a data-driven framework grounded in yield prediction and climate relevance ranking.

The Kansas High Plains was chosen as the testbed for this study because it represents one of the most agriculturally productive yet environmentally vulnerable regions in North America, characterized by a steep west-to-east precipitation gradient, recurrent droughts, and long-term depletion of the Ogallala Aquifer, one of the largest freshwater aquifers in the world. This study aims to rank a broad set of climatic features, capturing both seasonal variance through monthly measurements and long-term variance through annual measurements, to reflect temporal and environmental variability across Kansas. The objective of this study is to also assess the predictive capability of these ranked variables across irrigated and rainfed conditions within each agricultural district through ML-based regression models and forward selection. As water constraints intensify under climate variability over time, this study offers a scalable ML framework to identify climate-sensitive yield drivers, providing insights that can directly inform and guide crop management decisions. For example, if precipitation and temperature extremes consistently emerge as top predictors, that information can help refine when and how much to irrigate as Ogallala water supplies decline. Seasonal patterns in climate drivers can also guide when fields should be cut, so harvests avoid the worst periods of heat or drought stress. Likewise, knowing which factors are most predictive across districts can support the choice of more drought-tolerant cultivars in drier areas, or help anticipate when supplemental forage might be needed in high-risk years.

## Materials and methods

**Fig. 1:**
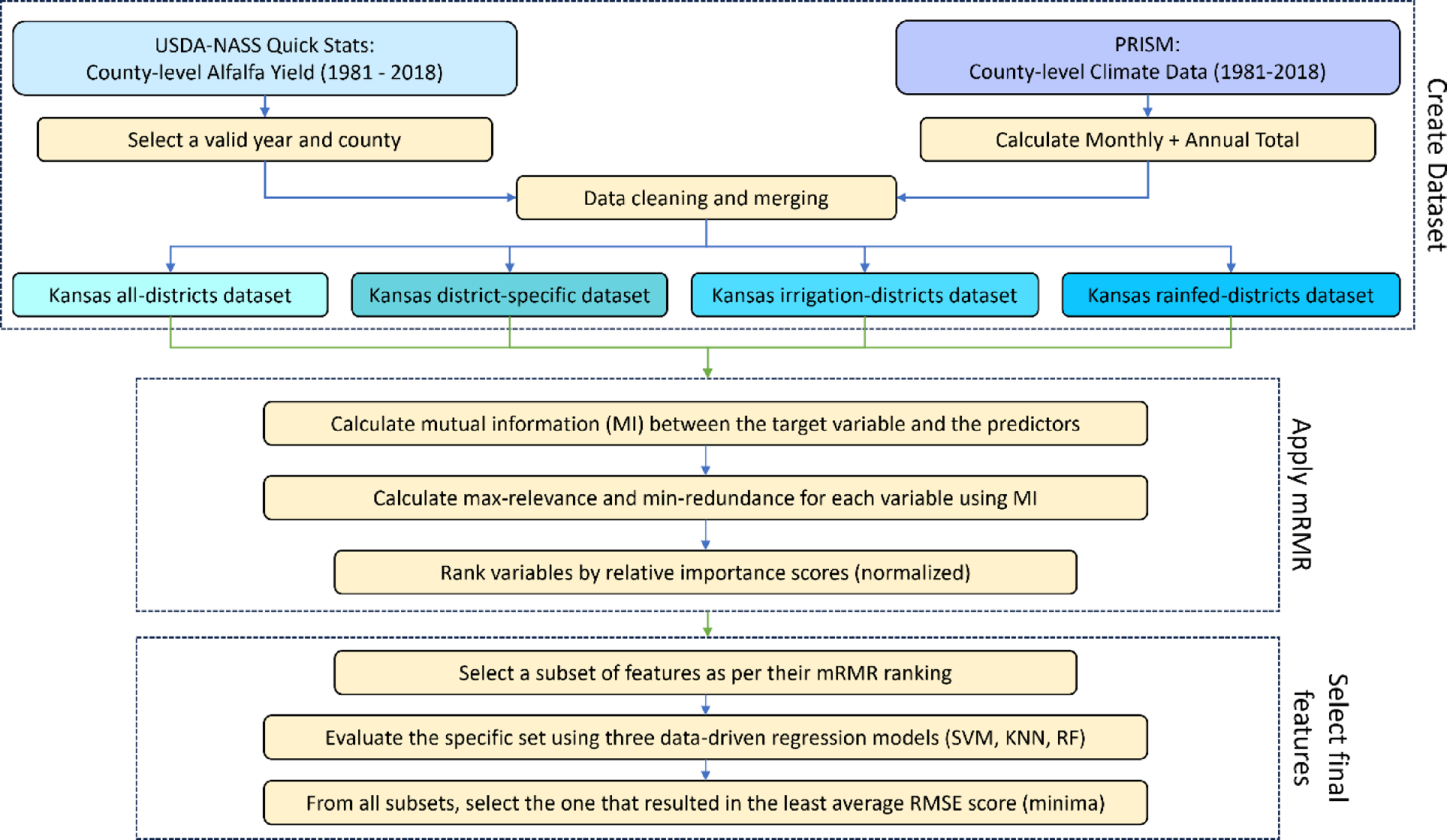
Overview of the methodology for selecting climate features most relevant to the alfalfa yield in Kansas using mRMR and regression models (SVM, KNN, RF) across four dataset types: all-districts, district-specific, irrigated, and rainfed. The final feature set for each dataset corresponds to the subset with the lowest observed RMSE (based on 5-fold cross validation) during forward selection.

This study investigated the relationship between climate variables and alfalfa yield across Kansas using a data-driven feature selection and evaluation framework (Fig. 1). County-level yield data from USDA-NASS^40^ and daily climate data from the PRISM Climate Group^41^ were collected for the years 1981–2018. The time series data for each county was generated on Jul 06, 2023. These county series were derived from the 4 km resolution PRISM AN81d gridded data within each county boundary^42^.

From the climate data, 117 features were computed to represent monthly and annual temperature, precipitation, humidity, and other climatic variables. To account for geographic and hydrological variability in yield response, the analysis was performed across four types of datasets: (i) all-districts combined (statewide), (ii) nine agricultural districts individually^22,37–39^, (iii) districts with primarily irrigated conditions, and (iv) rainfed districts. This classification reflects historical differences in groundwater access, precipitation patterns, and irrigation practices across the state and allowed us to investigate both regional patterns and the role of water availability in climate-yield relationships^43,44^.

To rank the agro-climatic variables as per their importance to alfalfa yield, the mRMR algorithm^31^ was implemented. Forward feature selection was then conducted by incrementally evaluating subsets of variables using three ML-based data-driven regression models: SVM^45,46^, KNN^47,48^, and RF^49,50^, respectively. Each model was trained and tested using 5-fold CV^51^, and performance was assessed using the regression RMSE^52^. For each dataset, the best observed feature set was selected based on the first local minimum in the moving average of the average RMSE across the three models. Figure 1 presents an overview of the methodological workflow.

### Location description

**Fig. 2:**
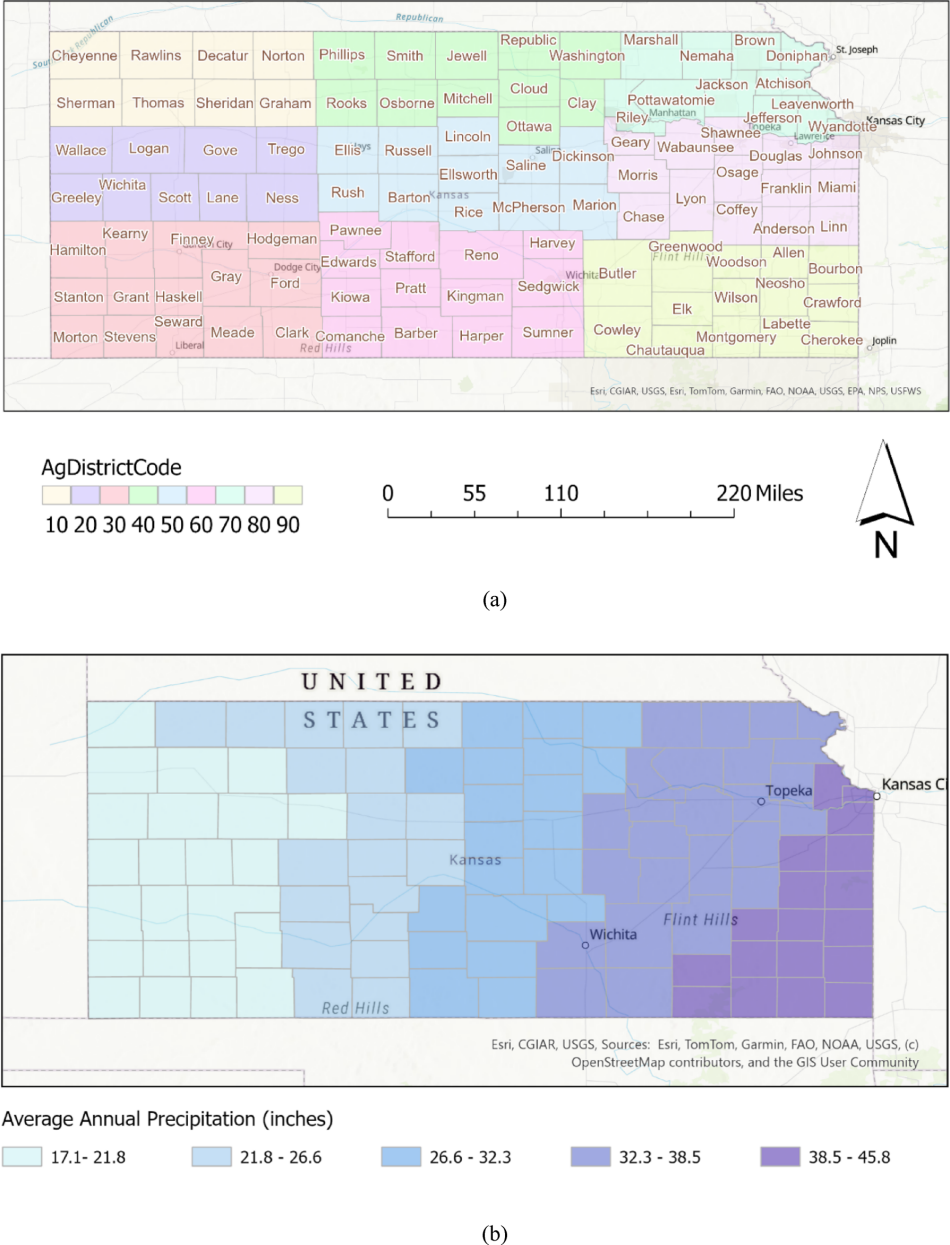
County-level map of Kansas showing (**a**) the nine agricultural districts color-coded by region. Based on water management practices, these nine districts were classified into two categories: six irrigation districts (10–60) and three rainfed districts (70–90); (**b**) Average annual total precipitation per county during the study period (1981–2018).

Alfalfa production in Kansas is shaped by significant climatic and hydrological gradients, which are reflected in its division into nine agricultural districts^37^. These districts, established by the USDA, align with county boundaries and are designed to capture regional differences in climate, topography, and water resource availability, providing a structured framework for agricultural data collection and analysis^38^. Figure 2a shows these nine districts, with counties labeled and color-coded by district to highlight regional distinctions. The average annual precipitation for each county during the study period are shown in Fig. 2b.

The east–west precipitation gradient across Kansas, ranging from 20 inches in the semi-arid west to 40 inches in the humid east^53^, is a major factor in differentiating the nine agricultural districts. This gradient directly influences water availability during the alfalfa growing season (April–October), with western districts often facing moderate to severe droughts that limit rainfed alfalfa production. The precipitation gradient is mirrored by an elevation rise from approximately 650 feet in the east to over 3900 feet in the west, influencing both temperature regimes and growing conditions^41^. Additionally, a north–south temperature gradient exists, with southern Kansas experiencing slightly warmer temperatures (about 58 °F average annual temperature) compared to the cooler northern regions (about 52 °F average annual temperature), further influencing the spatial variability in alfalfa growth conditions^54^.

To address the water differences, the nine districts were categorized into two sub-groups based on water management practices (Fig. 2): six districts with primarily irrigated conditions (districts 10–60) and three with rainfed conditions (districts 70–90). This classification was informed by data on groundwater usage, irrigation infrastructure, and precipitation patterns. In irrigated districts, access to groundwater resources, such as the Ogallala Aquifer, supports supplemental irrigation to mitigate drought stress. Conversely, rainfed districts rely solely on natural precipitation, making them more susceptible to yield reductions during dry periods^43^.

### Dataset description

#### Data sources

In this study, publicly available annual yield data of alfalfa from Kansas were combined with the corresponding weather parameters. The county-level yield data (tons/acre) for the years 1981 to 2018 were obtained from USDA-NASS^40^. The relevant climatic information were obtained from PRISM including precipitation, the minimum, mean, and maximum temperatures, the mean dew point temperature, and both the minimum and maximum values of vapor pressure deficit^41^. County-level yield estimates from USDA-NASS are derived from survey-based data and are subject to uncertainties associated with sampling error and nonresponse bias during reporting variability. These uncertainties are quantified by NASS using standard errors and coefficients of variation, although the magnitude of uncertainty varies depending on crop, region, and sample size.

#### Preprocessing

*Observations* The annual yield data from USDA-NASS^40^ were available for each year and for all counties and reported in tons per acre. However, starting from 2004, some of the counties collectively reported their average yield (109 in total). As this study focused more on analyzing the county-specific agro-climatic relationship, the combined county reports were removed while creating the dataset. As a result, we finally obtained a total of 3353 (84%) data points out of a total of 3990 observations for 105 Kansas counties over 38 years. The detailed county-specific missing years are reported in Table S1 in the supplementary material.

*Features* Seven daily weather parameters: precipitation (ppt), minimum temperature (tmin), average temperature (tmean), maximum temperature (tmax), average dew point temperature (tdmean), minimum vapor pressure deficit (vpdmin) and maximum vapor pressure deficit (vpdmax) from PRISM climate data^41^ were extracted for each county. The daily values were then combined to calculate the monthly and annual total for each of the parameters for each county from 1981 to 2018. This resulted in 13 features (12 monthly + 1 annual) for each of the seven climatic parameters obtained from PRISM (91 features in total for each of the 3353 points in our dataset). In addition, two additional parameters were calculated (both on a monthly and annual basis)—days with no precipitation (nopptdays) and Growing Degree Days (GDD).

*Days with no precipitation (nopptdays)* The number of days with more than 0 mm precipitation can significantly influence alfalfa growth, particularly during critical stages of development^15^. Especially during hot and sunny days, no precipitation can increase evapotranspiration demand while reducing soil moisture, limiting alfalfa growth and yield if irrigation is not available^55^. To quantify dryness, the number of days with no precipitation were calculated, both on a monthly and annual basis. A binary indicator variable, $\:{nopptday}_{n}=\left\{\mathrm{0,1}\right\}$ was defined, where, for each day * n* in the month or a year, 0 indicates no precipitation (0 mm) and 1 indicates precipitation above 0 mm. Then, the total number of ‘nopptdays’ over a one-month or one-year period was computed as follows.

1 $$\:nopptday{s}_{total}=\:\sum\:_{n=1}^{N}{nopptday}_{n}\:$$

where, * N* is the total number of days in the specified period (either 28/30/31 for a month or 365/366 for a year), and * n* represents each calendar day within that period.

*Total number of Growing Degree Days (GDD*_*total*_*)* Typically, the growing season for alfalfa is defined as the days with average temperature greater than 5 °C (base temperature for alfalfa production)^10,56^. In this study, for each month and year, the corresponding number of GDD was counted as the total number of days that resulted in a positive value for the following equation. Therefore, first, a binary indicator variable, $\:{GDD}_{n}=\left\{\mathrm{0,1}\right\}\:$was defined for each day, based on whether the corresponding GDD for that day is a positive number.

2 $$\:{GDD}_{n}\:=\left\{\begin{array}{c}1,\:\:\frac{tmax+tmin}{2}-tbase\ge\:0\\\:0,\:\:otherwise\end{array}\right.$$

where n represents each day between the years 1981 and 2018.

Then, the total number of days where $\:{GDD}_{n}=1$ was calculated over a monthly or annual period.

3 $$\:GD{D}_{total}=\:\sum\:_{n=1}^{N}{GDD}_{n}$$

where *N* is the total number of days in a month (for monthly GDD) or year (for annual GDD). Hence, in this study, including the ‘nopptdays’ and ‘GDD_total_^’^ parameters in addition to the PRISM climatic features, we used a total of 117 agro-climatic features. Table 1 summarizes these 117 agro-climatic features used in this study, including their variable names, temporal scale, units, and total feature count.

In this study, GDD was used as a climate-derived indicator of thermal accumulation and broad seasonal development potential. However, GDD does not directly represent cultivar-specific phenology or genetic differences in alfalfa growth habit. Alfalfa cultivars differ in fall dormancy, winter hardiness, regrowth rate after cutting, root architecture, drought tolerance, heat tolerance, and persistence, all of which can influence yield response under similar climatic conditions. Because the USDA-NASS county-level yield records used in this study do not include cultivar identity, dormancy class, stand age, genetic background, or cutting schedule, genetic effects could not be explicitly modeled. Therefore, GDD should be interpreted as an environmental proxy for thermal opportunity rather than a direct measure of genotype-specific developmental response.

**Table 1:** Summary of climate features used for agro-climatic feature ranking for alfalfa yield in Kansas (1981–2018).

Agro-climatic feature type	Variable name(s)	Data scale	Unit	No. of total features
Precipitation	ppt	Monthly	inches	12
		Annual	inches	1
Temperature	tmin, tmean, tmax	Monthly	°F	12 × 3 = 36
		Annual	°F	1 × 3 = 3
Dew Point Temperature	tdmean	Monthly	°F	12
		Annual	°F	1
Vapor Pressure Deficit	vpdmin, vpdmax	Monthly	hPa	12 × 2 = 24
		Annual	hPa	1 × 2 = 2
Days with no Precipitation	nopptdays	Monthly	days	12
		Annual	days	1
No. of Growing Degree Days	GDD_total_	Monthly	days	12
		Annual	days	1
**Total**	**-**	**-**	**-**	**117**

*Dataset partitioning* After filtering out the incomplete and missing data points and extracting 117 agro-climatic features, the collective dataset was partitioned into several categories based on geographical information and irrigation water management practices, as summarized in Table 2.

**Table 2:** Summary of dataset partitioning by agricultural district and water management practices in Kansas.

Dataset type	District(s) included	Irrigation water source	Number of data points
**All-Districts Combined**	**Districts 10–90**	**Both Irrigated and Rainfed**	**3353**
District-Specific	District 10	Irrigated	252
	District 20	Irrigated	252
	District 30	Irrigated	444
	District 40	Irrigated	356
	District 50	Irrigated	350
	District 60	Irrigated	431
	District 70	Rainfed	347
	District 80	Rainfed	465
	District 90	Rainfed	456
Irrigation Districts	Districts 10–60	Irrigated	2085
Rainfed Districts	Districts 70–90	Rainfed	1268

After the datasets were created, the mRMR feature selection algorithm was then applied separately to the all-districts combined dataset, individual district-specific datasets, and both irrigation and rainfed district groups to rank all the agro-climatic features as per their relevance to alfalfa yield under different environmental conditions.

### Feature selection process

Feature selection in this study was applied to identify a minimal subset of agro-climatic predictors that maximized the mutual information (MI) shared with observed alfalfa yield variability across spatial domains^52,57^. A total of 117 agro-climatic predictors were included, spanning annual and monthly metrics of temperature and moisture conditions. To control redundancy and retain predictors contributing meaningful information to yield variability, feature selection was implemented in two stages. First, predictors were ranked according to their relevance to yield and their redundancy with other features, assessed using correlation-based statistics derived from the combined datasets. Second, ranked features were evaluated using regression models within a forward selection framework, retaining those that improved predictive performance^58^.

Feature selection methods can generally be categorized into three main types: filter, wrapper, and embedded methods^59,60^. mRMR, a filter-based feature selection method, was used where each predictor relevance to observed yield variability was quantified using mutual information measures without involving a learning algorithm^51,61^. Wrapper and^52,60^ embedded methods perform feature selection during the model training process, as seen in decision trees or Lasso regression^59^. Because mRMR is a filter-based, model-independent method, it was selected to decouple feature ranking from downstream regression models. This enables a single, consistent feature ranking to be reused across different prediction pipelines, improving reproducibility and flexibility. In addition, its reliance on pairwise relevance and redundancy measures makes it computationally scalable for high-dimensional feature sets. Feature relevance scores reflect shared information with observed yield variability and do not imply effect size or causality. RMSE was calculated for feature subset to quantify model evaluation performance and was used to guide the selection of the subset that provided the most stable representation of observed yield variability.

#### Correlation analysis of predictors

To examine potential relationships between predictors and yield, both Pearson (linear) and Spearman (monotonic) correlations^52,62^ of all predictor variables with respect to yield were calculated (see details in the supplementary materials section). The corresponding correlation coefficients were in the range of -0.45 to 0.34 (Pearson) and − 0.44 to 0.34 (Spearman), with ‘tdmean_Jun_Total’ (total average dew point temperature in June) being the most negatively correlated (-0.45) variable and ‘vpdmax_May_Total’ (total maximum vapor pressure deficit in May) being the most positively correlated (0.34) variable (Fig. S1). The Pearson and Spearman coefficients were highly consistent (correlation = 0.9913), and the top 10 features selected based on both measures were identical.

The overall low correlation values indicate that most predictors exhibit weak or no monotonic relationship with yield, suggesting that simple linear models may be insufficient to capture the underlying relationships^63^. As a result, feature selection methods designed for linear models, such as LASSO, may not be appropriate for this dataset^51,52^. Furthermore, model-dependent approaches may restrict downstream modeling flexibility. Therefore, selecting a filter method (such as mRMR) provides the flexibility of incorporating it with any type of yield prediction and analysis model (both the deterministic and indeterministic ones)^57^.

To leverage this flexibility and capture potential non-linear relationships, we employed three ML-based regression algorithms (see more details in Sect.  2.3.4). These models are capable of learning complex patterns and interactions in the data without assuming linearity, which is particularly suitable given the weak correlations observed.

#### Minimum redundancy maximum relevance (mRMR) algorithm

The mRMR analysis was performed on the dataset to identify the most informative subset of agro-climatic predictors for alfalfa yield. This data-driven, model-free, filter-type algorithm selects features based on two criteria: (i) their individual relevance to the target variable (alfalfa yield) and (ii) their redundancy with other selected features. In this study, these criteria are measured using pairwise mutual information between each feature and the target yield, and among the features themselves.

The MI between any two continuous variables (e.g., an agro-climatic predictor and yield) quantifies their statistical interdependency and can be expressed as follows.

4 $$\:I\left(u,v\right)={\int\:}_{-\infty\:\:}^{\infty\:\:}{\int\:}_{-\infty\:}^{\infty\:\:}p\left(u,v\right)\mathrm{log}\frac{p\left(u,v\right)}{p\left(u\right)p\left(v\right)}dudv$$

where, $\:p\left(u\right)$ and $\:p\left(v\right)$ are the probability distribution of the variables $\:u\:$and $\:v\:$, whereas $\:p\left(u,v\right)$ is their joint probability distribution, respectively.

In this study, two categories of * u* and * v* were considered: (i) * u* is the target alfalfa yield and * v* all 117 agro-climatic variables and (ii) both * u* and * v* are pairwise combinations of all 117 agro-climatic variables.

A higher value of * I(u,v)* suggests stronger dependency, whereas, as the dependence between $\:u\:$and $\:v\:$ decreases, the $\:I\left(u,v\right)$ tends to zero more^64^. For example, the MI between ‘tdmean_Aug_Total’ (the total dew point temperature in August) and the alfalfa yield was calculated as 0.1355, which was much higher than the mean MI (0.0521) between yield and all variables. This indicates that ‘tdmean_Aug_Total’ has a high relevance with yield. On the other hand, ‘vpdmin_Jan_Total’ (the total minimum vapor pressure deficit in January) showed an MI of only 0.0088, indicating that ‘vpdmin_Jan_Total’ might not be strongly associated with yield variation, which aligns with limited crop growth in January. Intuitively, if $\:u\:$and $\:v\:$are completely independent, $\:I\left(u,v\right)=0$.

Using this concept of MI, mRMR aims to find the best set of $\:m\:\ge\:1\:$predictors (in descending order) that jointly have the largest dependence on the target $\:Y\:$as follows.

5 $$\:\underset{{S\:}\ni\:\:{X}^{i},{\:i}=1,\:\cdots\:{\:m}}{\mathrm{max}}D\left(S,Y\right)$$

where, $\:D\left(S,Y\right)$ is the dependency of $\:Y\:$on set of features, $\:S\:\ni\:\:{X}^{i},\:\left|S\right|\:=\:m$. The dependency itself is approximated as the mean value of the $\:I\left({X}^{i},Y\right)$, between $\:Y\:$ and each individual member of $\:S\:$as follows.

6 $$\:D\left(S,Y\right)=\frac{1}{m}{\sum\:}_{{X}^{i}\in\:S}^{\:}I\left({X}^{i},Y\right)$$

For example, if * S* a set of only 2 features, $\:S=\{\mathrm{ppt\_Apr\_Total},\:\mathrm{vpdmax\_May\_Total}\}$, and $\:I(\mathrm{ppt\_Apr\_Total},Y)=0.0598$, $\:I(\mathrm{vpdmax\_May\_Total},Y)=0.1108$, then $\:D(S,Y)=\:0.0853$. However, in this study, the mean MI was found to be 0.0521.

At the same time, the same concept of MI is used to minimize the size of $\:S\:$as follows to ensure that the smallest number of most effective features are selected.

7 $$\:\underset{{S\:}}{\mathrm{min}}R\left(S\right)=\frac{1}{{m}^{2}}{\sum\:}_{{X}^{i},{X}^{j}\in\:S}^{\:}I\left({X}^{i},\:{X}^{j}\right)$$

Therefore, combining the above two criteria (as shown in Eqs. 5 and 7), the mRMR algorithm optimizes the following objective function:

8 $$\:\underset{S}{\mathrm{max}}D\left(S,Y\right)\:-\:R\left(S\right)\:$$

To illustrate how the mRMR algorithm balanced relevance and redundancy, consider two features from the given dataset: ‘tdmean_annual_Total’ (the annual total dew point temperature) and ‘tdmean_Jun_Total’ (the total dew point temperature in June). Both were moderately relevant to alfalfa yield, with MI values of $\:I(\mathrm{tdmean\_annual\_Total},\:Y)=0.125$ and $\:I\left(\mathrm{tdmean\_Jun\_Total},\:Y\right)=0.130$, respectively. However, the redundancy between these two features was high with MI $\:I(\mathrm{tdmean\_annual\_Total},\:\mathrm{tdmean\_Jun\_Total})\:=\:0.593$.

In such cases, although ‘tdmean_annual_Total’ is informative, mRMR prioritized ‘tdmean_Jun_Total’ (which is slightly more relevant to the yield) and reduced the final score of ‘tdmean_annual_Total’ due to its strong redundancy with the selected feature (‘tdmean_Jun_Total’). Thus, a key strength of mRMR is that it favors selecting (top-ranking) a variety of predictors such that each of the predictors contributes independent information to the target variable, rather than duplicating information that is already captured.

Finding the optimal * S* is NP-hard^65^ i.e., it would require trying out exponential combinations of predictors to find the optimal set of them. Therefore, in this study, * S* was constructed using forward addition to reduce the computational complexity^31,33,64^.

#### Selection of the final features

The output of the mRMR algorithm for the climate dataset used in this study was a list of all predictors, ranked in order of their relative importance to the target variable. Hence, to select the best * n* predictors for each of the 12 datasets (Table 2) separately, we iterated through all the 117 features as per their mRMR ranking (starting from the most important to the least important one) and performed the following steps in each iteration^35,66^.

- *Step 1 (Cross validation)* for * i* (such that $\:i=\:1,\:2,\:\dots\:\:117$) predictors, create a subset, $\:{S}_{i}$ of the original dataset * S* and split it randomly into a 5-fold CV^67^. Thus, for each data partition, four folds were used to train the prediction models (described below), and the 5th fold was used for testing. This process was repeated five times. Thus, the 12 datasets, the number of datapoints per fold were 670 for the all-districts datasets; 50, 50, 88, 71, 70, 86, 69, 93, 91 for Districts 10, 20, 30, 40, 50, 60, 70, 80, and 90 respectively; and 417 and 253 for the irrigation and rainfed datasets, respectively. 
- *Step 2 (Evaluation models)* for each set of * i* predictors, $\:{S}_{i}$, evaluate its relevance to the yield using three widely used ML-based prediction models^56^—support vector machines (SVM)^45,46^, K-nearest neighbors (KNN)^47,48^, and random forest (RF)^49,50,68^, respectively. The RMSE score^52^ was used as an evaluation metric for the model prediction performances^56^. Each of these prediction models was trained using four folds, and the test RMSE was calculated for the 5th fold. This process was repeated five times, and for each model, the average of these five simulations was considered as its corresponding evaluation score (see Fig. S6 and Table 3 for details). Then, for the set of predictors $\:{S}_{i}$, the final score was calculated as an average of the three prediction models (denoted as ‘Average Value’ in the figures).
- *Step 3 (First local minimum of the moving average)* The moving average of the final scores (average value) was calculated and all the local minima were calculated. Then, the first local minimum (occurring with the least number of predictors) was selected as the ‘best RMSE’ and the corresponding $\:{S}_{i}$ was selected as the set of best features.

Using Brute force method that checks every possible combination of features, a total of $\:1.66\times\:{10}^{35}$ model evaluations are needed for each dataset which is not possible to simulate with a feasible time. In contrast, for the presented forward feature selection method, three models, 117 different sets of features, and 5-fold CV for each dataset were used. And the total number of model evaluations was $\:3\times\:\:117\times\:\:5\:=\:1755$. Hence, the best set of features were reported based on 21,060 simulations for all 12 datasets (Table 2).

### Implementation details

The proposed feature selection method was implemented in MATLAB^69^. All outputs, including the datasets, were later converted into a ‘.csv’ file format. The mRMR algorithm was implemented using the ‘fsrmrmr’ function from Statistics and Machine Learning Toolbox™^70^.

For selection of the final features, several functions from Statistics and Machine Learning Toolbox™^70^ were used. For the 5-fold CV, the ‘cvpartition’ function was used (along with ‘training’ and ‘test’ functions). For the SVM and RF models, the functions ‘fitrsvm’ (with the ‘KernelFunction’) specified as ‘rbf’ representing Radial Basis Function^71^ and ‘fitrensemble’ (with the ‘method’ specified as ‘bag’, representing Bootstrap Aggregating)^72^ were used, respectively. However, for KNN, we implemented ‘kNNeighborsRegressor’ function^73^ with Euclidean distance specified. The predict function was used for all prediction purposes.

The yield data and some agro-climatic features (temperature, precipitation) were displayed using the surface plot operator (surf), and the mRMR results were plotted using the bar graph operator (bar) on MATLAB. For reproducibility of the results, we used the default value of the ‘rng’ (Mersenne Twister generator with seed 0) function on MATLAB.

**Fig. 3:**
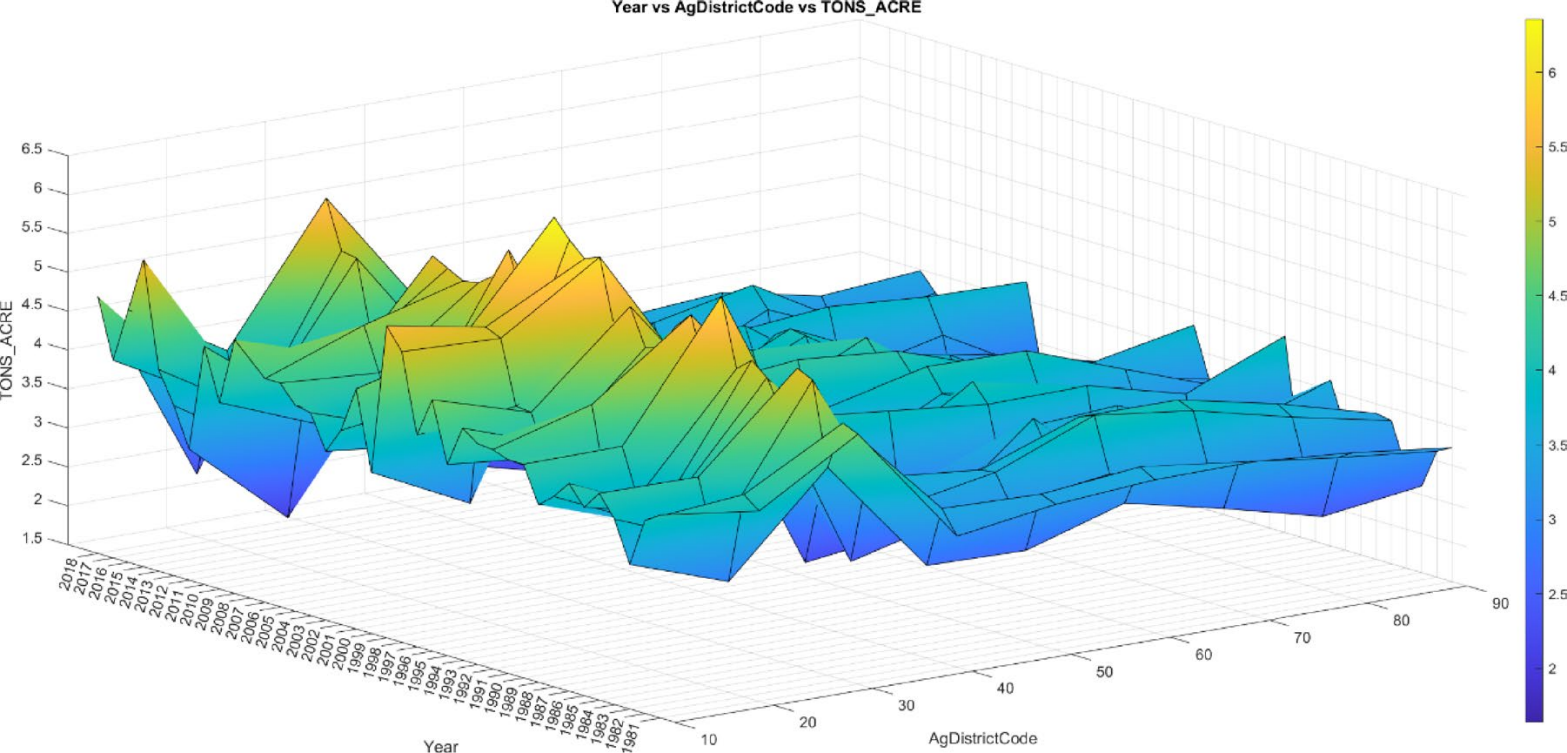
Average yield for all counties in each district (districts 10‒90) for the years 1981—2018, obtained from USDA-NASS. The x-axis represents the years, the y-axis indicates the agricultural districts (10–90), and the z-axis depicts the average yield (tons/acre).

For visualization purposes only (e.g., Fig. 3), missing yield data for certain years and districts were filled using the 1-D data linear interpolation operation (default in ‘interp1’) in MATLAB, to avoid discontinuities in the plots. This interpolation was performed temporally within each district. internal gaps were estimated from the line drawn between the nearest non-missing years, while edge cases (e.g., missing values in 1981 or 2018) were replaced with the immediate adjacent non-missing year. These interpolated values were not used in the analysis.

## Results and discussion

### Kansas agro-climatic data analysis

#### Yield analysis of nine agricultural districts in Kansas (1981–2018)

Figure 3 shows the average alfalfa yield from 1981 to 2018. The yield data analysis reveals notable disparities in alfalfa yield per acre in districts 10, 20, and 30, where yield consistently exceeds 4 tons/acre. District 30 stands out with yields approaching close to 6 tons/acre in 1990 and 1999 and more than 6 tons/acre in 1998, 2000, and 2001. In contrast, rainfed districts (70‒90) consistently demonstrate lower yield production, typically ranging between 1.8 and 4.1 tons/acre throughout the studied period.

During drought occurrences, alfalfa production experiences a corresponding decrease^10^. Notably, Kansas faced a severe drought in 2012^74^, contributing to fluctuating yields over the years. The highest recorded yield, slightly exceeding 6.35 tons/acre, occurred in 2001 (district 30), whereas the lowest yield, just under 1.64 tons/acre, was reported in 2011 (district 60). Based on temperature and precipitation, this study reveals that alfalfa dry matter yield in western Kansas surpassed that of eastern Kansas.

The alfalfa yield differs according to the district, and there was a dramatic difference between districts 10‒30 and districts 40‒90. This observation, despite gaps in the 2008 dataset, challenges the conventional view that precipitation is the dominant driver of alfalfa growth and instead highlights the predominant role of irrigation in districts 10–60^43^. A comparison of the same counties between 2010 (non-drought) and 2012 (severe drought) confirmed that alfalfa yield increased with higher irrigation inputs during both years, with the effect being more pronounced under drought conditions^75,76^.

#### Temperature and precipitation analysis of agricultural districts (1981-2018)

**Fig. 4:**
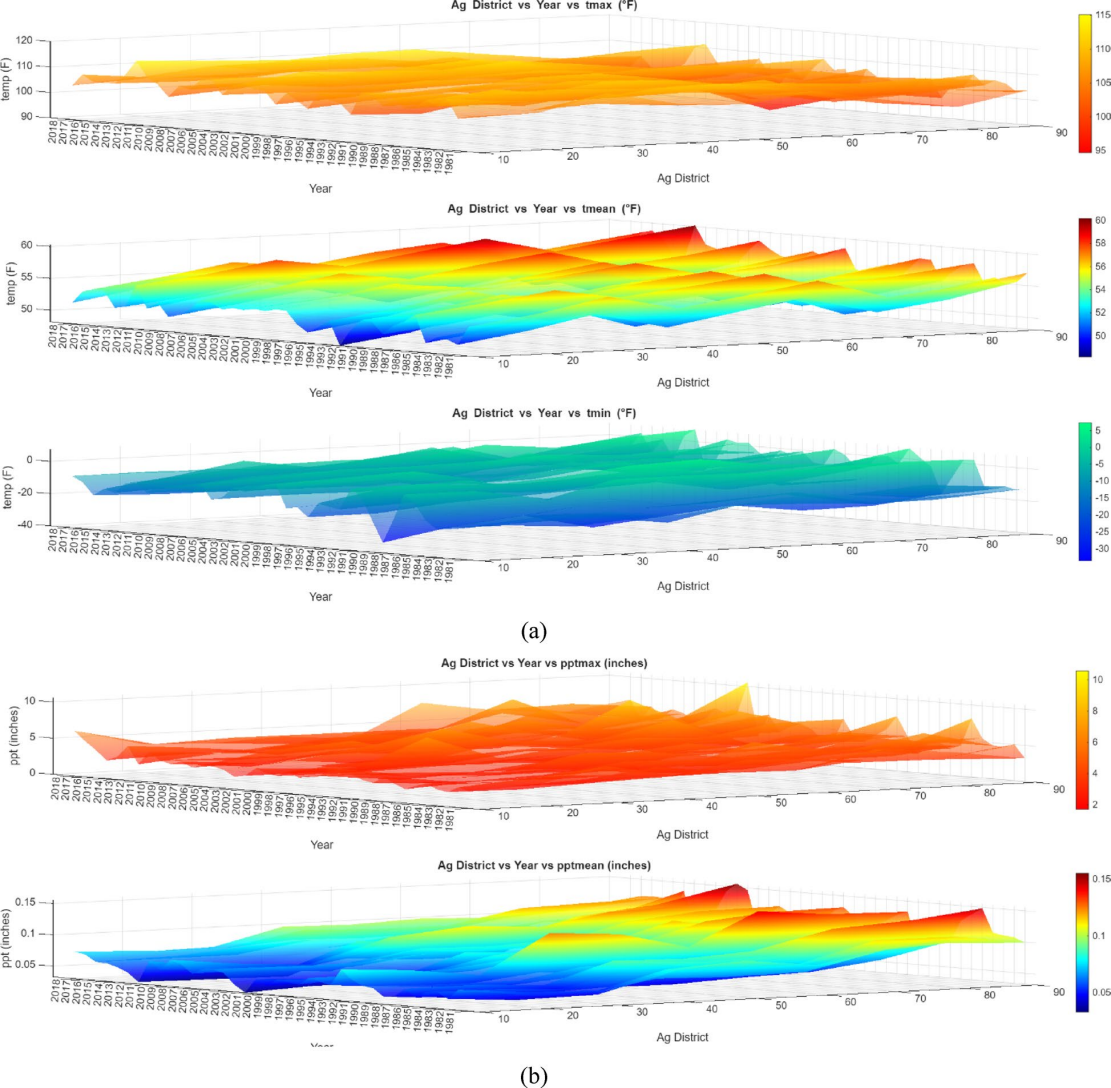
Maximum (top), mean (middle), and minimum (bottom) daily temperature (**a**), and maximum (top) and mean (bottom) daily precipitation (**b**), across counties in districts 10–90 for the period 1981–2018.

For all nine agricultural districts in Kansas (10‒90) and years of interest for this study (1981-2018), Fig. 4a shows the daily maximum (tmax), average (tmean) and minimum (tmin) temperature. The daily maximum temperature predominantly falls within the range of 95–115 °F across the years (Fig. 4). Conversely, daily average temperature exhibits a variation, mostly between 49 and 59 °F, with district 90 reaching close to 60.2 °F in 2012 and district 10 dropping to slightly over 48.15 °F in 1993. Furthermore, specific years like 1981, 1986-87, 1990-92, 1998-2001, 2006, and 2011-12 exhibit elevated daily average temperature readings (close to 60 °F), particularly in districts 30, 50, 60, and 90. In contrast, daily minimum temperature displays greater variability (mostly within the range of -30 °F to 5 °F), with the highest being 7.3 °F in district 90 in 2012 and the lowest being − 33.6 °F in district 10 in 1989.

Figure 4b presents the daily maximum (pptmax) and average (pptmean) precipitation recorded in inches for districts 10 to 90 and years 1981 to 2018. Daily average precipitation seems to maintain a gradient of precipitation across the districts, with a range of mostly 0.04‒0.15 inches. The western districts (10‒30) experienced lower precipitation (mostly below 0.07 inches with some exceptions in the years 1993, 1996, 2004-2006, 2009, 2011, 2015, and 2017-2018). Moreover, the year 1993 shows comparatively higher precipitation of at least 0.02 inches than the average for all districts, and an exceptionally high range of precipitation (0.11-0.12 inches) in some of the Central region (districts 40 and 50).

The eastern districts (70‒90, also the rainfed districts) show a comparatively higher range of precipitation, mostly above 0.1 inches. For these districts, some of the years (such as 1985-1987, 1992-1993, 1997-1999, 2007-2009, and some areas after 2013) show a higher precipitation amount, mostly in the range of 0.13 to 0.15 inches. In contrast, certain years, such as 1988, 1991, 1994, 2000, 2002, and 2012, experienced a lower range of precipitation, typically around 0.035 to 0.05 inches. However, the year 2006 presented an intriguing scenario where precipitation seemed uniformly distributed across all districts, exhibiting a narrow variation within the range of 0.062 to 0.083 inches. Remarkably, this resulted in comparatively higher precipitation for the typically low precipitation western districts and lower precipitation for the high precipitation eastern districts during that year.

Moreover, it is important to highlight the existence of several days without any reported precipitation, identified as no precipitation days (nopptdays) and accounted for as a distinct parameter in this study. Overall, considering all days in a year, including those with and without precipitation, the irrigation districts (10‒60) averaged daily precipitation of 0.07 inches, whereas the rainfed districts (70‒90) averaged 0.11 inches, which is 0.04 inches higher than the irrigation districts.

### Model-based feature selection performance evaluation

The 117 agro-climatic features used in this study (described in the dataset description section) were ranked in order of their importance by the mRMR algorithm individually for each of the 12 datasets (Table 2). Next, each of the feature subsets (obtained in a forward feature selection manner) was evaluated using three ML algorithms—SVM, KNN, and RF, using the default model parameters. These include an RBF kernel with standardized predictors for SVM, k = 4 with Euclidean distance and distance-weighted averaging for KNN, bootstrap aggregation with 100 decision trees and default tree-growing parameters for RF. No hyperparameter tuning was done, as the goal was feature relevance, not predictive optimization^77^. The same forward selection process was repeated for the features ranked in order of their importance by the Pearson’s correlation for the Kansas all-districts dataset, which served as a baseline approach.

**Fig. 5:**
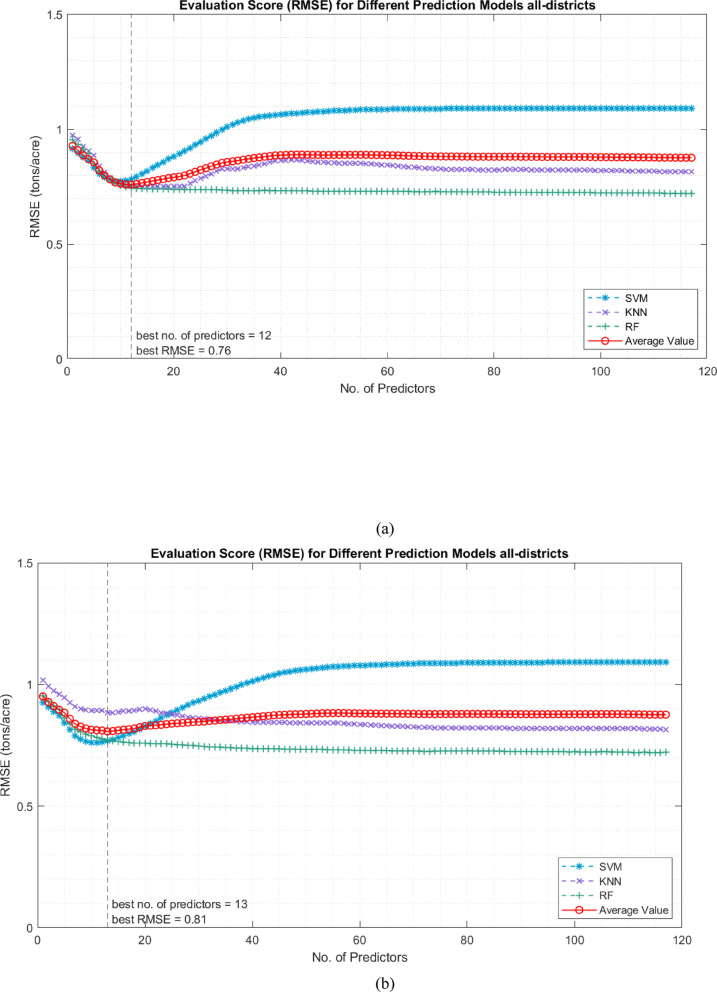
Average RMSE (tons/acre) for all three ML-based evaluation models (SVM, KNN, RF) on Kansas all-districts dataset using (**a**) mRMR-ranked features and (**b**) Pearson-ranked features.

The performance evaluation results for all-districts datasets are shown in Fig. 5 for (a) mRMR-ranking and (b) Pearson-ranking. Here, the x-axis represents the number of predictors forming the evaluated feature subset. For example, ‘No. of Predictors = 1’ denotes the highest-ranked feature by mRMR (Fig. 5a) and Pearson (Fig. 5b) for that dataset. Similarly, ‘No. of Predictors = 2’ denotes the top two highest-scoring features, and so forth, up to ‘No. of Predictors = 117’, which includes all available features for the dataset. The y-axis depicts the corresponding test RMSE of the 5th fold (as described in the ‘selection of the final features’ subsection) for the three ML models, along with the average of their RMSE (denoted by the red ‘Average Value’ line).

As the mRMR-ranked subfigure shows, with increasing number of features, the evaluation performance used to guide feature subset selection increases (leading to a lower average RMSE) up until 12 predictors with an average RMSE of 0.76 tons/acre by SVM, KNN, and RF. Beyond this point, with an increasing number of predictors, the RF model performance continues to improve marginally by less than 0.01 tons/acre in total, reaching a plateau. But at the same time, SVM performance starts deteriorating in between 8 and 10 features up to 35–37 features before reaching a plateau at 1.05 tons/acre RMSE at first and then slowly increasing up to 1.09 tons/acre. A similar behavior is observed in the case of KNN, whose RMSE goes as low as 0.75 tons/acre in between 12 and 22 features and then slowly increasing up to 0.86 tons/acre around 40 features, followed by decreasing and stabilizing at 0.83 tons/acre at around 70 features.

From the Pearson-ranked subfigure, the progression of RMSE across increasing numbers of predictors followed a broadly similar trend to that of the mRMR-ranked features. The optimal average RMSE was obtained with the top 13 Pearson-ranked predictors, yielding 0.81 tons/acre, which is 0.05 tons/acre higher than the mRMR-ranked optimal subset (12 features, 0.76 tons/acre). Model-specific behaviors revealed further contrasts between the two ranking strategies. Under Pearson’s ranking, KNN exhibited consistently higher RMSE across nearly all subset sizes compared to the mRMR case, which substantially contributed to the higher overall error. While SVM and RF performances were more comparable between the two methods, both showed slightly elevated RMSE under Pearson ranking. Notably, RF only surpassed the mRMR performance when more than 58 predictors were included, which is far beyond the mRMR optimum of 12 features and Pearson optimum of 13 features.

This decline in prediction accuracy with increasing features illustrates the curse of dimensionality, where sparse high-dimensional data limits model generalization. This effect was evident under both mRMR and Pearson rankings. SVM and KNN, being distance-based models, struggled beyond 10–15 features due to insufficient sample-to-feature ratios. In contrast, RF remained stable, likely due to its ensemble learning approach. Therefore, as the combined performance of the three ML models reaches its first local minimum at 12 (stops improving before they start deteriorating), the final set of features chosen from the all-districts dataset is the top-scored 12 features by mRMR, as shown in Table 3. From the table, using the proposed approach, the number of selected features varies from 7 (district 80) to 21 (district 10) for different datasets, with a corresponding RMSE of 0.52 to 1.14 tons/acre, respectively. The difference in the number of selected features suggests that district 10 faces a complex environment in which irrigation management interacts closely with localized weather extremes, requiring more features to achieve lower RMSE. In contrast, district 80 is a rainfed system, where alfalfa yield is dominated by fewer climatic drivers, making additional features redundant. Essentially, while more data points in district 80 increase statistical confidence, the underlying simplicity of rainfed production allows the model to capture the variance with only 7 variables.

**Table 3:** Number of selected features (as per their mRMR ranking) and the corresponding RMSE score for all 12 datasets for alfalfa production in Kansas.

Dataset	Number of selected features	RMSE (tons/acre)
All-districts	12	0.76
District 10	21	0.71
District 20	9	0.92
District 30	15	1.14
District 40	12	0.52
District 50	11	0.60
District 60	11	0.72
District 70	9	0.63
District 80	7	0.61
District 90	10	0.59
Irrigation districts (10—60)	10	0.86
Rainfed districts (70—90)	10	0.62

### mRMR selected features

During the summer months, particularly in late July to August, alfalfa growth may be depressed due to the occurrence of higher temperatures in the region. Especially, summer slump is associated with high temperatures, compounded by increased humidity and elevated night temperatures during the summer monsoon^78,79^. Consequently, an increase in respiration rates during warm weather led to decreased net photosynthesis and yield^80^.

Precipitation patterns vary across districts, and they increase from district 10 to district 90 (west to east of Kansas). Western Kansas has been experiencing moderate to exceptional drought for decades from April to October, coinciding with the alfalfa growing season^10,44^. While the eastern region receives more suitable precipitation levels, the western region faces challenges due to insufficient precipitation in Kansas. The factors affecting alfalfa production include the amount of sunlight, length of growing season, temperature, and drought intensity, with precipitation distribution during the growing period crucial for growth, regrowth, and overall yield^10^. Despite varying moisture requirements based on climate and soil conditions, optimal alfalfa cultivation conditions typically necessitate 19.7 to 45.3 inches of water, except for districts 10–30. The water demand for alfalfa production varies, with approximately 4 to 5 inches of water required for each ton/acre of hay production^81^. A high no precipitation day correlates with increased sunshine exposure, indicating more days with higher sun intensity. In irrigated conditions where water needs are met, a day without precipitation can facilitate continuous alfalfa growth, potentially boosting yield. Conversely, in rainfed conditions where moisture is limited, precipitation becomes crucial for alfalfa growth, leading to a positive response to precipitation but a negative impact on the number of no precipitation days variable.

#### mRMR selected variables for the Kansas all-districts dataset

**Fig. 6:**
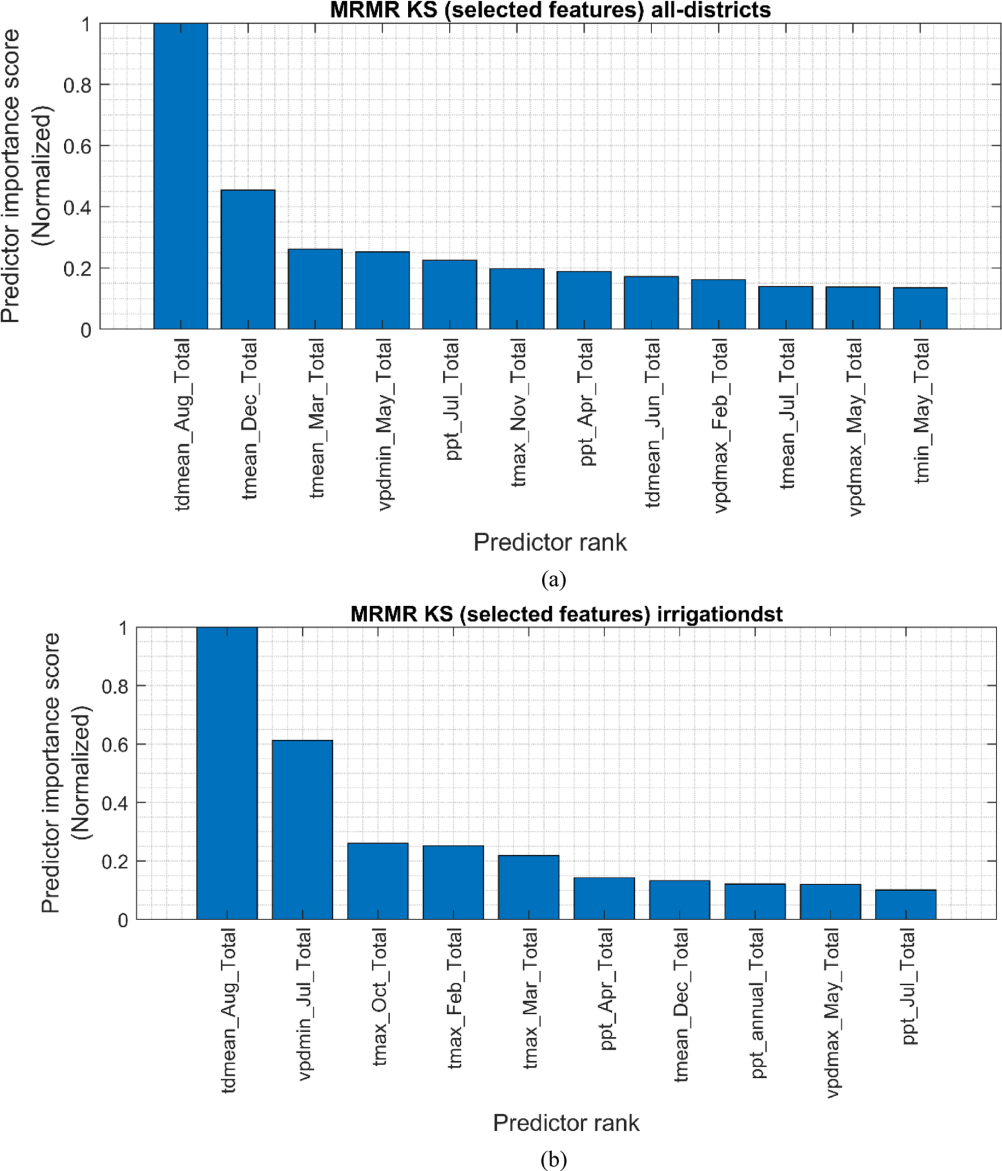

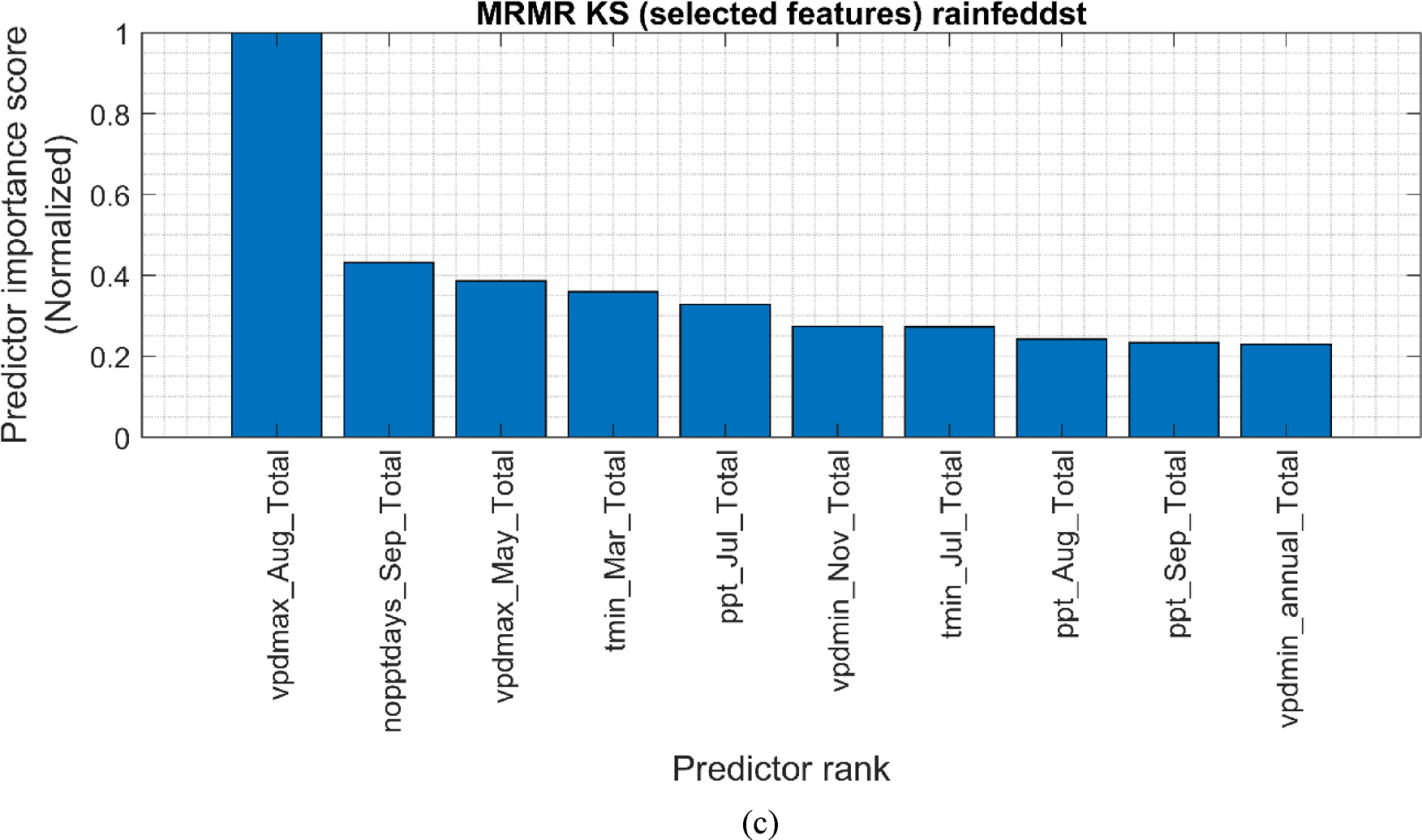
mRMR selected climate variables affecting alfalfa yield for Kansas all-districts (**a**), districts under irrigated (**b**) and rainfed (**c**) condition in Kansas.

Across all districts in Kansas, twelve variables were selected for their effect on alfalfa production, revealing that the mean dew point temperature of August (Tdmean_Aug) emerged as the most influential factor (Fig. 6a). The mean dew point temperature of August (Tdmean_Aug) showed a negative relationship with alfalfa yield, which could be due to high temperatures and humidity reducing the evapotranspirative cooling effect from the crop resulting in crop stress (Fig. S8a). An increased precipitation in July had significant positive correlation with alfalfa production which is understandable due to the high temperatures and water need in the summer (Fig. S8a). Considering the variability in climate among western and eastern Kansas, it is required to understand and address the factors influencing alfalfa yield separately by the districts.

#### mRMR selected variables for the Kansas district-specific datasets

The variables with the highest correlation were number of no precipitation days of September (nopptday_sep), number of no precipitation days of November (nonpptday_Nov), Growing Degree Days of April (GDD_Apr), annual precipitation (ppt_annual), precipitation of July (ppt_Jul), mean temperature of July (tmean_Jul), precipitation of July (ppt_Jul), minimum VPD of August (VPDmin_Aug), and maximum VPD of July (VPDmax_Jul) in districts 10, 20, 30, 40, 50, 60, 70, 80, and 90, respectively. This shows that the variables exerting the highest impact varied across districts, with distinct patterns emerging. Districts 10, 20, 40, 50, and 70 showed a prevalence of the precipitation variable. Meanwhile, variables related to growing degree days and vapor pressure deficit were selected for districts 30, 60, 80, and 90. This difference suggests that the cultivation technique of alfalfa varied among the districts. Specifically, districts 10–60 exhibited a focus on temperature and precipitation variables, while districts 70–90 prioritized precipitation and evapotranspiration variables. Given that irrigation is prevalent in districts 10–60, whereas rainfed conditions prevail in districts 70–90, it underscores the need for distinct analyses tailored to these regions.

From 1890 to 2015, the western region of Kansas was observed to be moisture-limited, which gradually increased due to rising temperatures, increased evaporation rates, low precipitation, and more frost-free days. Meanwhile, the eastern region of Kansas recorded a gradual increase in moisture levels during the same period. In districts 10 to 60, irrigation management shows a statistically negative association with annual precipitation and yield variation related to annual precipitation with a correlation of -0.23 (*p* < 0.05) (Fig. S8b). The maximum temperature in the starting of the season and ending of the growing season such as March and October recorded a positive correlation on the alfalfa production. It is plausible that in the irrigated districts (10 to 60), total irrigation water applied per acre during low-precipitation years exceeded the actual crop evapotranspirative demand (Fig. S8b). This over-application may have reduced root-zone oxygen availability, increased nutrient leaching, or disrupted osmotic balance, all of which could negatively affect alfalfa growth. Consequently, higher yields in years with higher maximum temperature in the March will help with warming up soil temperatures which will help to break the winter dormancy^82^, and ending of the season in October, it will help in setting optimal time for cutting in dry days in October which will affect subsequent year’s alfalfa production^13^. On the other hand, the districts from 70 to 90, where rainfed conditions prevail, precipitation positively impacted production as it had significant positive correlation (*p* < 0.05) (Fig. S8c). It was found that an increase in temperature negatively affected production, likely due to increased moisture stress resulting from higher VPD and Tdmean as temperature increases.

#### mRMR selected variables for the Kansas irrigation-districts dataset

In irrigated conditions, the dew point temperature of August (Tdmean_Aug) and minimum vapor pressure deficit of July (VPDmin_Jul) had a higher negative correlation than other variables (Figs. 6b and S8(b)). However, most of the variables related to temperature were selected. In particular, the influence related to moisture was high. The dew point temperature of August (Tdmean_Aug) and minimum vapor pressure deficit of July (VPDmin_Jul) appeared as the most informative variables associated with yield variation, but these are inversely related, as dew point temperature and vapor pressure deficit were indices of humidity and dryness, respectively. So, if the higher August humidity is known to coincide with reduced summer-season yield potential, consistent with the observed negative associations^26^. The minimum vapor pressure deficit of July was the second-most influential variable under the irrigated conditions. In July, Kansas experiences the highest air temperatures, coupled with dry conditions that pose challenges for meeting alfalfa’s water requirements. While irrigation offers a solution to mitigate the challenge, prolonged exposure to high temperatures exceeding 100 °F, combined with standing water for more than 24 h, can lead to burn damage known as scald injury^26^. Therefore, in irrigated conditions, it was found that many variables related to temperature were selected, indicating that moisture stress is alleviated through irrigation practices^83^.

#### mRMR selected variables for the Kansas rainfed-districts dataset

In the rainfed condition, maximum vapor pressure deficit of August (VPDmax_Aug) emerged as the most influential factor by negatively affecting alfalfa yield, with most selected variables revolving around vapor pressure deficit, number of no precipitation days, and precipitation parameters (Figs. 6c and S8(c)). Alfalfa, known for its high moisture requirements, exhibits a strong dependency to humidity and precipitation levels^9,84,85^. Thus, making maximum vapor pressure deficit of August (VPDmax_Aug), a critical determinant under rainfed conditions, particularly due to the dry conditions prevalent in August. Despite the significance of precipitation, it was found that variables, namely total precipitation of August (ppt_Aug_total), and total precipitation of September (ppt_Sep_total), had a low positive impact in the rainfed condition. In rainfed conditions, the normal precipitation levels recorded (1991-2022) were over 29.0 inches^10^, thus satisfying the water requirements for alfalfa growth.

#### mRMR selected key features in different agro-climatic regions in Kansas

In irrigation conditions, the selected variables were in the order temperature, precipitation, vapor pressure deficit, and dew point temperature (Fig. 6b). However, in rainfed conditions the order of variables is different, namely-vapor pressure deficit, number of precipitation days, temperature, and precipitation (Fig. 6c). Overlapping variables between irrigated and rainfed conditions included maximum vapor pressure deficit of May (VPDmax_May) and precipitation of July (ppt_Jul) (Fig. 6b, c). Maximum vapor pressure deficit of May had significant positive impact in yield (*p* < 0.05) under irrigated conditions, whereas non-significant negative correlation (*p* > 0.05) with alfalfa yield under rainfed conditions (Fig. S8b, c). The maximum vapor pressure deficit of May (VPDmax_May) is selected likely due to the timing of the first alfalfa harvest in May, when the crop demands ample moisture post-harvest^22^. Meanwhile, precipitation of July (ppt_Jul) had significant positive correlation with alfalfa yield in both the conditions because the water demand of alfalfa has increased due to the weather in Kansas, which is characterized by high temperature and low precipitation in July.

The months selected for both irrigation and rainfed conditions overlapped notably in March, May, July, and August (Fig. 6b, c). The temperature in March was critical for both irrigated and rainfed conditions, highlighting its importance among climatic features. May is crucial as the regrowth of alfalfa begins at this period, and it is dependent on the temperature. On the other hand, in the month of May, alfalfa is harvested, and the vapor pressure deficit variable is considered to be impactful to alfalfa production as it is selected under both rainfed and irrigated conditions because the demand for moisture increases after harvest. July was selected as a precipitation variable under both conditions, which suggests an increased moisture demand for alfalfa due to high temperature. Lastly, August featured a mean dew point temperature under irrigation, maximum vapor pressure deficit, and precipitation under rainfed, indicating varied factors influencing alfalfa growth during this period.

Several studies have revealed that water is a major yield-limiting factor in alfalfa production^10,86–89^. While precipitation was initially expected to be the primary influencer in Kansas, it appears to have less impact compared to variables associated with vapor pressure deficit and dew point temperature related to drought and humidity, respectively. This distinction is critical because vapor pressure deficit and dew point act as direct indicators of atmospheric demand and moisture availability, both of which exert continuous influence on crop water status regardless of management. Unlike precipitation, whose effect varies depending on irrigation inputs, VPD and dew point consistently explained yield variability across both irrigated and rainfed systems. Their stability as predictors highlights their value for crop yield modeling, where management-independent drivers are needed. These findings indicate that atmospheric moisture variables consistently carry high informational relevance to observed yield variability, suggesting they warrant further investigation in future alfalfa modeling studies. VPD and dew point are inherently less tied to local management practices. This makes them more transferable predictors across irrigated and rainfed systems, and even across regions with different irrigation technologies or soil types.

The vapor pressure deficit suitable for alfalfa is known to be 3 kPa^90^. Within the timeframe of this study, VPD levels above this threshold appeared intermittently, typically in roughly every alternate year. The same was observed in the Northwest and Midwest regions as the number of 1-day periods above 9 kPa increased by 30–40 days in the late century. It was reported that the dew point temperature significantly increased in the range of 33 to 35 °F in the Midwest region of Kansas^91^. Especially in the Central Midwest, the increase in dew point temperature is highest. Dew point extremes are expected to increase in late summer, adding to climate uncertainty in Kansas^92–98^.

Under the irrigated conditions, variables primarily linked to humidity and dryness (mean dew point temperature, maximum vapor pressure deficit, and minimum vapor pressure deficit), alongside temperature, were selected, with mean dew point temperature appearing as one of the top-ranked variables by mRMR and exhibiting a negative association with yield based on the correlation analysis. Conversely, rainfed conditions were characterized by variables related to precipitation or dryness (maximum and minimum vapor pressure deficit), with evapotranspiration variables appearing among the most informative predictors in mRMR rankings. Notably, August emerged as the pivotal month influencing both conditions, given its highest impact on alfalfa cultivation due to elevated temperatures and dryness in Kansas.

#### mRMR selected top 10 features in comparison to Pearson’s and Spearman’s correlation coefficients

For the all-districts datasets, the top 10 features by both Pearson’s and Spearman’s are: ‘tdmean_Jun_Total’, ‘tdmean_annual_Total’, ‘tmin_Jul_Total’, ‘tdmean_Aug_Total’, ‘tdmean_Apr_Total’, ‘tdmean_Jul_Total’, ‘tmin_Jun_Total’, ‘tdmean_May_Total’, ‘tmin_annual_Total’, ‘tmin_Aug_Total’. In contrast, the top 10 features by mRMR algorithm represented a more diverse set of predictors, including not only dew point, but also average temperatures, VPD, both maximum and minimum temperatures, and precipitation: ‘tdmean_Aug_Total’, ‘tmean_Dec_Total’, ‘tmean_Mar_Total’, ‘vpdmin_May_Total’, ‘ppt_Jul_Total’, ‘tmax_Nov_Total’, ‘ppt_Apr_Total’, ‘tdmean_Jun_Total’, ‘vpdmax_Feb_Total’, ‘tmean_Jul_Total’, ‘vpdmax_May_Total’, ‘tmin_May_Total’.

This comparison highlights an important methodological distinction. Correlation-based rankings capture only the strongest marginal associations between individual predictors and yield, which often results in the repeated selection of highly related variables. In contrast, mRMR explicitly balances relevance and redundancy, favoring variables that contribute complementary and non-redundant information to the prediction task. As a result, the feature set identified by mRMR is more heterogeneous and compact, yet ultimately more predictive.

In alfalfa production, it is meaningful to include VPD as a primary variable because it is a direct driver of transpiration and plant water stress, representing the true atmospheric demand for water. This is far more insightful than relying on individual correlations with temperature, which oversimplify the complex plant-environment interaction. An increase in mean dew point in August is statistically correlated with lower yields, but interpreting this correlation requires understanding the underlying physiological cause that is high humidity (low VPD). When the air is saturated, the VPD is low, which reduces the plant’s ability to cool itself through transpiration. The VPD synthesizes the combined effect of temperature and humidity into a single metric that directly reflects the biophysical stress on the plant, providing a more reliable and actionable understanding of yield variability than simple correlation-based rankings.

#### mRMR limitations and future work

While the mRMR algorithm effectively identified climate variables most relevant to alfalfa yield while minimizing redundancy, several limitations should be considered. First, the algorithm relies on pairwise MI, which can be sensitive to data quality, sample size, and variable discretization. District-specific datasets with fewer observations may lead to less stable feature rankings. Second, complex interactions or nonlinear dependencies between variables were only accounted for through MI, not explicitly modeled during feature selection. Future work could explore whether model-specific tuning improves selection sensitivity. Future work may explore other feature selection methods and broader geographic applications. Another limitation of this study is the lack of genetic and genotype-by-environment effects; GDD serves only as a general proxy and does not capture cultivar-specific phenology or stress responses due to dataset constraints. Alfalfa yield is influenced by traits such as dormancy, regrowth, and stress tolerance, which are not represented in the current framework. Future work should couple this ML approach with process-based models like DSSAT-Alfalfa to explicitly integrate genetic responses with climate drivers.

## Conclusions

This study identified climatic drivers of alfalfa yield using mRMR and ML-based regression. At first, the mRMR algorithm was used to rank all features from most to least important. Next, three ML predictors (SVM, KNN, and RF) were used to evaluate yield variability using 5-fold CV for each dataset with an incremental number of features. Finally, the best set of features was selected based on their corresponding average test RMSE.

To account for climatic and irrigation management differences across the state, the analysis was conducted separately for irrigated and rainfed conditions, in addition to a district-specific as well as a statewide combined dataset. In this regard, variables related to August moisture stress, including mean dew point temperature (Tdmean_Aug) and maximum vapor pressure deficit (VPDmax_Aug), were ranked as top predictors across datasets. This highlights the relevance of late-summer conditions with alfalfa yield in Kansas.

Under irrigated conditions, the selected variables were temperature, precipitation, vapor pressure deficit, and dew point temperature. However, in rainfed conditions, the selected variables were vapor pressure deficit, precipitation, temperature, and number of no precipitation days. Across all datasets, climatic variables, including VPD and precipitation of certain months, especially March (associated with the onset of vegetative growth), May (around the first harvest), and July–August (peak summer stress period), were identified as the key predictors, emphasizing their importance for alfalfa production.

This data-driven framework helps uncover yield drivers and can be adapted to other crops and regions. Our data-driven approach highlights the importance of management-independent factors like VPD and dew point and offer a useful basis for future climate-yield modeling studies to sustain alfalfa production, providing a transferable framework for resilient crop modeling in drought-prone regions. Future work could extend this framework by integrating remote sensing-derived variables, soil properties, and process-based crop models to further improve mechanistic understanding and scalability across diverse agro-ecological systems.

## Supplementary Information

Below is the link to the electronic supplementary material.

Supplementary Material 1

## Data Availability

The datasets analyzed in this study are publicly available from the following sources: Alfalfa yield data: USDA National Agricultural Statistics Service (NASS). Climate data: PRISM Climate Group (https://prism.oregonstate.edu/). Additional regional precipitation and temperature data: Kansas Mesonet (https://mesonet.k-state.edu/). All data and analysis scripts used in this study are available at the GitHub repository linked below: https://github.com/BMW-lab-MSU/Alfalfa-Yield-ML-Kansas.

## Abbreviations

USDA-NASS: USDA National Agricultural Statistics Service
PRISM: Parameter elevation Regression on Independent Slopes Model
RH: Relative humidity
VPD: Vapor pressure deficit
GDD: Growing degree days
ML: Machine learning
mRMR: Minimum redundancy maximum relevance
MI: Mutual information
SVM: Support vector machines
KNN: K-nearest neighbors
RF: Random forest
CV: Cross validation
RMSE: Root mean squared error
