# Supplementary material for: Data-driven and machine learning framework for Alfalfa yield response to long-term climate variability in the Kansas High Plains (USA)
Source: Sci Rep. 2026 Jun 4;16:25610. doi: 10.1038/s41598-026-55545-2 (PMC13478387; doi:10.1038/s41598-026-55545-2)
Supplement: Supplementary file 1 [file 41598_2026_55545_MOESM1_ESM.docx]

# Supplement Section 1: Pearson’s and Spearman’s Correlation Analyses:

## Pearson’s correlation:

Pearson’s (standard) correlation coefficient[1] between a set of predictor variables ($X^{k}$) and the target variable ($Y$) can be calculated using the following equation.

$$C^{k}=cor\left( X^{k},Y \right)=\frac{\sum_{n=1}^{N} \left( x_{n}^{k} -\overline{x^{k}} \right)\left( y_{n} -\overline{y} \right)}{\sqrt{\sum_{n=1}^{N} \left( x_{n}^{k} -\overline{x^{k}} \right)^{2}} \sqrt{\sum_{n=1}^{N} \left( y_{n} -\overline{y} \right)^{2}}}$$

Here, $x_{n}^{k} \in X^{k}$ is the value of the $k^{th}$ predictor for each of the datapoint, $y_{n} \in Y$, respectively. Each correlation coefficient $C^{k}$ is a measure of the linear dependency of the target variable $Y$with the respective predictor, $X^{k}$^1^. Usually, for $\left| C^{k} \right|<0.6$, $Y$is not considered to be strongly linearly dependent on $X^{k}$^2^. However, for the Kansas yield dataset, the correlation coefficients were all in the range of -0.45‒0.34, as shown in Fig. S1. Here, ‘tdmean_Jun_Total’ (total average dew point temperature in June) was determined as the most negatively correlated variable and ‘vpdmax_May_Total’ (total maximum vapor pressure deficit in May) was the most positively correlated variable, respectively.

From the figure, most precipitation (ppt) features (except for July) show negative correlations with alfalfa yield. A similar trend is observed between minimum temperature (tmin), average dew point temperature (tdmean) and average temperature (tmean) except for ‘tmean’ in January and February. The opposite trend is observed for maximum vapor pressure deficit (vpdmax), Days with no precipitation (nopptdays) except for ‘nopptdays’ in July and the maximum temperature (tmax) except for ‘tmax’ in August. Both minimum vapor pressure deficit (vpdmin) and total number of growing degree days (GDD_total_) show a very small correlation with respect to yield.

(a)

(b)

Fig. S1. (a) Pearson’s and (b) Spearman’s correlation coefficients of all 117 predictors with respect to yield for the Kansas all-districts dataset.

We also calculated the Pearson’s and Spearman’s correlation coefficients between the features themselves to see whether there was interdependency in between the features themselves, as shown in Fig. S2.

(a)

(b)

Fig. S2. (a) Pearson’s and (b) Spearman’s correlation coefficients between all 117 predictors for the Kansas all-districts dataset.

As a result of the correlation analysis, most of the variables were low correlated with alfalfa dry matter yield. Variables with low correlations are as follows: ‘VPDmax_Dec.’, ‘Tmin_Jul.~Aug.’, ‘Tmean_annual’, ‘TDmean_Apr.~Aug.’ under irrigation conditions, and ‘Tmin_Aug.’, ‘Tmean_Jul.~Aug.’, ‘Tmax_Jul.~Aug.’, ‘VPDmean_annual’, ‘VPDmin_Jul.~Aug.’, ‘VPDmax_annual’, and Jul.~Aug under rainfed conditions (Fig. S3).

In particular, the rainfed condition selected variables were temperature and VPD variables, and most of the periods were July to August (Fig. S4).

Also, the results of comparing the correlation between irrigated and rainfed conditions are as followed. In irrigated conditions, ‘VPDmax’, ‘Tmean’ and ‘Tmax’, ‘TDmean’, and ‘nopptdays’ showed a positive relationship with the alfalfa dry matter yield. Rainfed condition showed a positive relationship between ‘ppt’ (summer season) and ‘TDmean’ with alfalfa dry matter yield. Under this result, precipitation, VPD, and ‘nopptdays’ showed opposite trends between irrigated and rainfed condition.

Fig. S3. Correlation values (both positive and negative) between all variables and the target variable, yield (tons/acre), irrigation districts, ppt threshold 0 mm (about 0 in).

Fig. S4. Correlation values (both positive and negative) between all variables and the target variable, yield (tons/acre), rainfed districts, ppt threshold 0 mm (about 0 in).

## Spearman’s correlation:

Spearman’s rank correlation coefficient between a set of predictor variables ($X^{k}$) and the target variable ($Y$) is defined as the Pearson correlation of the rank-transformed values of $X^{k}$ and $Y$.

$$R^{k}=cor\left( rank(X^{k}),rank(Y) \right)$$

Equivalently, when there are no tied ranks, Spearman’s correlation can be expressed as:

$$R^{k}=1-\frac{6\sum_{n=1}^{N} d_{n}^{2}}{N(N^{2}-1)}$$

where $d_{n}= rank(x_{n}^{k}),rank(y_{n})$ is the difference between the ranks of the $n^{th}$ observation, and $N$ is the total number of samples^3^.

Each coefficient $R^{k}$ measures the strength of a monotonic relationship between the predictor $X^{k}$ and the target variable $Y$, regardless of whether the dependency is linear or non-linear. Unlike Pearson’s correlation, Spearman’s correlation is less sensitive to outliers and can capture predictor-wise non-linear monotonic associations.

For the Kansas yield dataset, the correlation coefficients were all in the range of -0.435‒0.336 (very similar to Pearson’s), as shown in Fig. S1.

## Comparing Pearson’s ($C^{k}$) and Spearman’s ($R^{k})$ correlation:

$C^{k}$and $R^{k}$ showed very similar data distributions, with a correlation of 0.9913 between them. The detailed statistical summary of these variables are as follows:

$C^{k}$: mean=-0.0552, median=-0.0730, min=-0.4499, max=0.3435
$R^{k}$_y: mean=-0.0682, median=-0.0859, min=-0.4352, max=0.3359

The top 10 features selected based on both correlation measures were identical:

'tdmean_Jun_Total', 'tdmean_annual_Total', 'tmin_Jul_Total', 'tdmean_Aug_Total', 'tdmean_Apr_Total', 'tdmean_Jul_Total', 'tmin_Jun_Total', 'tdmean_May_Total', 'tmin_annual_Total', 'tmin_Aug_Total'

Supplement Section 2: Performance Evaluation of mRMR-ranked and Pearson’s-ranked features:

## Features to data points per fold ratio for each dataset:

Fig. S5. Ratio of number of datapoints per fold with respect to the number of features for the 12 Kansas alfalfa yield datasets.

# Supplement Section 3: district-specific mRMR selected features:

Fig. S6. mRMR selected climate variables affecting alfalfa yield for districts 10‒90 in Kansas district-specific dataset.

# Supplement Section 4: Missing yield data per county in the USDA-NAAS dataset (1981-2018):

A total of 637 entries (~15.96%) were missing out of 3990 entries (38 years, 105 counties) from the USDA-NAAS yield data.

**Table S1: Missing yield data per county in the USDA-NAAS dataset**

| County | Missing Years |
| --- | --- |
| allen | 2009 2016 2017 2018 |
| anderson | 2009 2011 2012 2013 2016 2017 2018 |
| atchison | 2005 2006 2008 2009 2011 2012 2013 2014 2015 2016 |
| barber | 2008 2013 2016 2018 |
| barton | 2016 2018 |
| bourbon | 2009 2012 2013 2015 |
| brown | 2013 2015 2016 |
| butler | 2008 2009 2010 2014 2015 2016 2018 |
| chase | 2008 2009 2012 2017 2018 |
| chautauqua | 2008 2009 2012 2013 2015 2016 2017 2018 |
| cherokee | 2009 2010 2011 2012 2013 2014 2015 2016 2018 |
| cheyenne | 2008 2011 2016 |
| clark | 2008 2011 2016 |
| clay | 2011 2012 2013 2014 2015 2016 2017 2018 |
| cloud | 2008 2009 2014 2015 2016 2018 |
| coffey | 2008 2009 2012 2013 2016 |
| comanche | 2008 |
| cowley | 2008 2009 2013 2014 2015 2016 2017 2018 |
| crawford | 2008 2009 2010 2013 2014 2016 |
| decatur | 2008 2012 2014 2015 2016 2017 2018 |
| dickinson | 2013 2014 2015 2016 2017 2018 |
| doniphan | 2008 2009 2015 2016 2017 2018 |
| douglas | 2009 2012 2017 2018 |
| edwards | 2008 2015 2016 2017 |
| elk | 2008 2009 2015 2017 2018 |
| ellis | 2008 2009 2010 2012 2013 2014 2015 2016 2017 2018 |
| ellsworth | 2008 2009 2010 2012 2014 2015 2018 |
| finney | 2008 2009 2010 2011 2014 2015 2016 2017 2018 |
| ford | 2008 2017 |
| franklin | 2008 2009 2017 2018 |
| geary | 2008 2016 2018 |
| gove | 2008 2009 2010 2018 |
| graham | 2008 2010 2011 2015 2016 |
| grant | 2008 2009 2013 |
| gray | 2008 2012 2013 2014 2015 2016 2018 |
| greeley | 2003 2004 2005 2006 2007 2008 2009 2010 2011 2012 2013 2014 2015 2016 2017 2018 |
| greenwood | 2014 2016 |
| hamilton | 2008 2015 2016 2017 |
| harper | 2008 2011 2012 2014 2015 2016 2017 2018 |
| harvey | 2009 2014 |
| haskell | 2008 2010 2011 2015 2017 2018 |
| hodgeman | 2008 2009 2010 2011 2013 2014 2015 2016 2018 |
| jackson | 2009 2014 2015 |
| jefferson | 2009 2013 2014 2015 2016 2018 |
| jewell | 2008 2011 2013 2014 2015 2016 2017 2018 |
| johnson | 2008 2009 2010 2013 2014 2015 2016 2017 2018 |
| kearny | 2008 2012 2013 2017 |
| kingman | 2008 2009 2011 2015 2018 |
| kiowa | 2008 2009 2011 2012 2015 2016 2018 |
| labette | 2009 2010 2012 2013 2015 2016 |
| lane | 2003 2004 2006 2007 2008 2013 2015 2016 2017 |
| leavenworth | 2009 2010 2013 |
| lincoln | 2008 2009 2014 2015 2016 2017 2018 |
| linn | 2008 2010 2011 2013 2015 2017 2018 |
| logan | 2008 2009 2010 2011 2012 2013 2014 2015 2016 2017 2018 |
| lyon | 2009 2016 |
| marion | 2015 2016 2018 |
| marshall | 2012 2013 2014 2015 2016 2017 2018 |
| mcpherson | 2013 2015 2016 2017 2018 |
| meade | 2008 2010 2018 |
| miami | 2008 2009 2010 2011 2012 2016 2017 |
| mitchell | 2008 2016 |
| montgomery | 2009 2010 |
| morris | 2008 2014 2015 |
| morton | 2003 2005 2006 2008 2009 2010 2011 2012 2013 2016 2017 2018 |
| nemaha | 2009 2011 2012 2013 2015 2016 2017 2018 |
| neosho | 2009 2012 2016 2017 |
| ness | 2008 2009 2011 2013 2014 2015 2016 2017 2018 |
| norton | 2008 2012 2015 2016 2018 |
| osage | 2009 2010 |
| osborne | 2008 2009 2012 2018 |
| ottawa | 2014 2015 2016 2017 2018 |
| pawnee | 2015 2018 |
| phillips | 2008 2014 2015 2016 2017 2018 |
| pottawatomie | 2015 2016 2017 2018 |
| pratt | 2008 2009 2010 2011 2012 2013 2014 2015 2016 2018 |
| rawlins | 2008 2009 2012 2013 2014 2015 2016 2017 2018 |
| reno | 2015 2016 2017 2018 |
| republic | 2011 2012 2013 2016 2017 2018 |
| rice | 2008 2011 2012 2013 2014 2015 2016 2017 2018 |
| riley | 2008 2009 2012 2014 2015 2016 2017 2018 |
| rooks | 2008 2011 2012 2013 2015 2016 |
| rush | 2008 2010 2011 2012 2013 2014 2015 2016 2017 2018 |
| russell | 2015 2016 2017 2018 |
| saline | 2014 2015 2016 2017 2018 |
| scott | 2008 2009 2010 2011 2012 2013 2014 2015 2016 2017 2018 |
| sedgwick | 2013 2014 2015 2017 2018 |
| seward | 2004 2005 2008 2018 |
| shawnee | 2008 2009 2013 2014 2015 2016 |
| sheridan | 2008 2009 2014 2015 2016 2017 2018 |
| sherman | 2008 2011 2012 2013 2015 2016 2018 |
| smith | 2013 2014 2015 2016 2017 |
| stafford | 2008 2010 2011 2012 2013 2014 2015 2016 2017 2018 |
| stanton | 2003 2004 2005 2006 2008 2009 2010 2011 2012 2013 2014 2016 2017 |
| stevens | 2005 2008 2009 2012 2014 2015 2016 2017 2018 |
| sumner | 2013 |
| thomas | 2008 2009 2010 2011 2013 2015 2016 2017 2018 |
| trego | 2008 2009 2011 2012 2013 2015 2016 2017 2018 |
| wabaunsee | 2008 2014 2015 |
| wallace | 2008 2011 2012 2013 2014 2015 2016 2017 2018 |
| washington | 2013 2014 2015 2016 2017 2018 |
| wichita | 2005 2008 2009 2010 2011 2012 2013 2014 2015 2016 2017 2018 |
| wilson | 2008 2009 2011 2012 2014 2015 2017 2018 |
| woodson | 2012 2013 2015 |
| wyandotte | 2005 2006 2008 2009 2010 2011 2012 2013 2014 2015 2016 2017 2018 |

# Supplement Section 5: Top 10 features selected by mRMR and Pearson’s:

Fig. S7. mRMR selected (top) and Pearson selected (bottom) top 10 climate variables affecting alfalfa yield for Kansas all-districts dataset.

# Supplement Section 6: Pairwise relationship of yield and mRMR selected top 10 features in irrigated, rainfed, and all districts

(a)

(b)

(c)

Fig. S8. Pair-wise relationship of alfalfa yield with top 10 selected features by mRMR for Kansas all-districts (a), districts under irrigated (b), and rainfed (c) condition in Kansas.

References

1.	James, G., Witten, D., Hastie, T. & Tibshirani, R. *An Introduction to Statistical Learning*. vol. 103 (Springer, New York, NY, 2013).

2.	Ooi, L., Ng, K.-L., Heng, W.-C., Chua, Y.-F. & Lim, Y.-H. Human resource practices and employee engagement: A study among academicians in Malaysian private higher education institutions. *International Journal of Management Studies* **29**, 71–100 (2022).

3.	Ali Abd Al-Hameed, K. Spearman’s correlation coefficient in statistical analysis. *International Journal of Nonlinear Analysis and Applications* **13**, 3249–3255 (2022).
